# HumanNet v2: human gene networks for disease research

**DOI:** 10.1093/nar/gky1126

**Published:** 2018-11-10

**Authors:** Sohyun Hwang, Chan Yeong Kim, Sunmo Yang, Eiru Kim, Traver Hart, Edward M Marcotte, Insuk Lee

**Affiliations:** 1 Department of Biotechnology, College of Life Science and Biotechnology, Yonsei University, Seoul 03722, Korea; 2 Center for Systems and Synthetic Biology, Institute for Cellular and Molecular Biology, University of Texas, Austin, TX 78712, USA; 3 Department of Biomedical Science, College of Life Science, CHA University, Seongnam-si 13496, Korea; 4 Department of Bioinformatics and Computational Biology, The University of Texas MD Anderson Cancer Center, Houston, TX USA; 5 Department of Molecular Biosciences, University of Texas at Austin, TX 78712, USA

## Abstract

Human gene networks have proven useful in many aspects of disease research, with numerous network-based strategies developed for generating hypotheses about gene-disease-drug associations. The ability to predict and organize genes most relevant to a specific disease has proven especially important. We previously developed a human functional gene network, HumanNet, by integrating diverse types of omics data using Bayesian statistics framework and demonstrated its ability to retrieve disease genes. Here, we present HumanNet v2 (http://www.inetbio.org/humannet), a database of human gene networks, which was updated by incorporating new data types, extending data sources and improving network inference algorithms. HumanNet now comprises a hierarchy of human gene networks, allowing for more flexible incorporation of network information into studies. HumanNet performs well in ranking disease-linked gene sets with minimal literature-dependent biases. We observe that incorporating model organisms’ protein–protein interactions does not markedly improve disease gene predictions, suggesting that many of the disease gene associations are now captured directly in human-derived datasets. With an improved interactive user interface for disease network analysis, we expect HumanNet will be a useful resource for network medicine.

## INTRODUCTION

Human gene networks have been widely used to investigate genetic factors of diseases and therapeutic targets (1). Gene networks can also augment disease genomics information derived from expression profiles (2–4), whole exome sequencing (5,6) and genome-wide association studies (GWAS) (7,8) for the discovery of disease-associated genes. Edges of the gene networks may represent diverse types of associations between genes which can be mapped by both experimental and computational methods. Because appropriately integrating interaction information from diverse sources can improve the breadth and accuracy of a network, many integrated human gene networks have been developed and a variety of topological analysis algorithms have been applied to generate new hypotheses about gene-disease-drug associations.

We previously developed an integrated human functional gene network, HumanNet, and demonstrated its capability of disease gene predictions (9). In order to construct the network, we inferred functional associations between human genes from protein–protein interactions (PPI), co-citation of human genes across PubMed abstracts, co-occurrence of protein domains, co-expression of genes across samples and genomic context associations. In addition, interactions between evolutionarily conserved proteins of model organisms were transferred to the human gene network. Those networks, inferred from different types of data, were evaluated and integrated using a Bayesian statistical framework. Since the first release of HumanNet, the amount of publicly available omics data has increased substantially and network inference algorithms have also improved significantly, and thus we expected that updating HumanNet could provide a greatly enhanced resource for network medicine.

**Figure 1.**
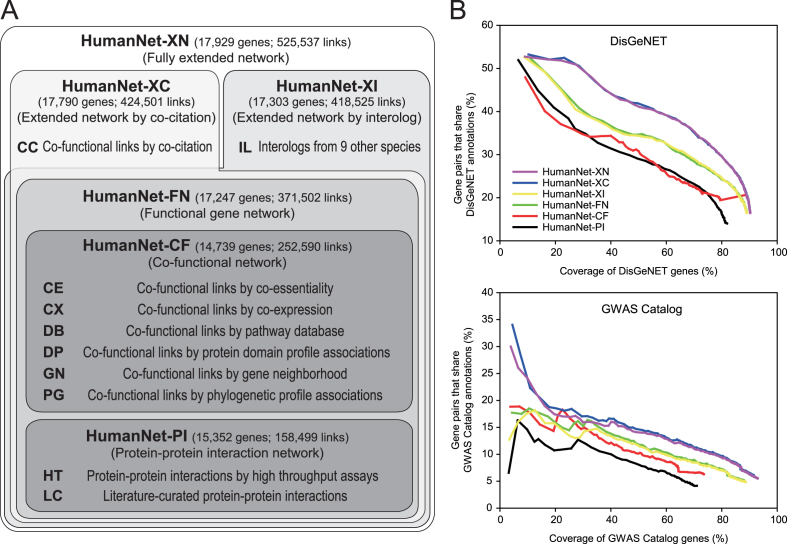
(**A**) Overview of the four level hierarchy of human gene networks in the HumanNet database. (**B**) Assessment of the six human gene networks at different levels of the hierarchy, based on measuring the precision of identifying gene pairs linked to the same human diseases (defined by DisGeNET or GWAS catalog with timestamp filtration) as a function of the coverage of the database genes.

In this report, we present HumanNet v2, which offers substantial performance improvements over v1, especially for the disease gene predictions. A new feature of the updated HumanNet is a four level inclusive hierarchy of the human gene networks: the first level has two networks, HumanNet-PI comprising human-derived PPIs and HumanNet-CF based on co-functional links inferred from various types of genomics data; the integration of HumanNet-PI and HumanNet-CF produces the second level network HumanNet-FN which is an integrated functional gene network; the third level has two extended functional networks by either co-citation (HumanNet-XC) or interologs (10) from other species (HumanNet-XI); and the fourth level network is the fully extended network (HumanNet-XN) that contains all above functional links (Figure 1A).

We benchmarked each of the networks for their ability to prioritize disease-linked gene sets with two different network-based algorithms. We observed HumanNet-XC and HumanNet-XN to have equally good or better performance than STRING v10.5 (11) and significantly better performance than other integrated human gene networks such as ConsensusPathDB (CPDB) (12), GIANT (7), GeneMANIA (13) and FunCoup (14). Time-stamped benchmarking strategy demonstrated that the improvements in performance of HumanNet extended beyond the incorporation of literature-based information. Interestingly, while we offer networks extended by IL for completeness, we observed no gains in disease gene prediction quality by their incorporation, suggesting that data measured directly in humans has reached a high level of predictive power for the disease gene network. Users can download edge information of various human gene networks and perform disease gene predictions and disease network analysis via a highly interactive user interface on the HumanNet web server (www.inetbio.org/humannet).

## NETWORK DATABASE IMPROVEMENT

### Four-level inclusive hierarchy of human gene networks

To provide flexibility in utilizing the network's information for various purposes, we designed HumanNet v2 with a four-level inclusive hierarchy of human gene networks comprising networks based on 10 distinct types of data (Figure 1A and Supplemental Table 1). The previous version of HumanNet was constructed based on only functional associations between genes, which can be supported by various types of biological data. The PPI assay was a traditional approach for mapping the functional associations between genes. Human gene networks based on only PPIs generally have a limited network coverage, because there are many functional associations that are not mediated by physical interactions between proteins. However, PPI networks have advantages in terms of the mechanistic interpretation of disease-associated mutations (15). Therefore, we decided to maintain a human gene network based on only PPIs separately as one of the first-level networks, HumanNet-PI, which contains 158 499 links among 15 352 genes, based on PPIs by high-throughput assays (HT) and literature-curated PPIs (LC).

In contrast to the PPI network, functional gene networks can be supported by diverse types of data (16), including PPIs. Despite lacking mechanistic information for the network links due to the broad edge definition, the typically high comprehensiveness of functional gene networks provides advantages in terms of generating functional hypotheses. We inferred co-functional associations between genes from six additional types of data: co-essentiality (CE) (17), co-expression (CX) (18), associations by pathway database (DB), associations between protein domain profiles (DP) (19), associations by gene neighborhood (GN) (20) and associations between phylogenetic profiles (PG) (21). Network inference methods for each type of data are described in the Supplemental Methods. We integrated the six co-functional gene networks to generate another first-level network based on only inferred co-functional links from omics data, HumanNet-CF that contains 14 739 genes and 252 590 links. Integration of these two first-level networks produces the second-level network HumanNet-FN, an integrated functional gene network that contains 17 247 genes and 371 502 links.

Two networks at third-level were constructed based on the extended information of the functional associations by either co-citations (CC) across approximately 300 000 full-text articles of PubMed Central (HumanNet-XC) or interologs (IL) transferred from nine other species (HumanNet-XI). Co-citation made a significant contribution to the mapping of functional associations for several human gene networks, including HumanNet and STRING. However, the functional network by co-citation may cause biased benchmarking performance for disease gene discovery, because benchmarking data are also based on the literature. Some users may want to exclude the influence of co-citation during disease gene predictions. Therefore, we decided to maintain a human gene network extended by co-citation data separately. HumanNet-XC contains 17 790 genes and 424 501 links. In contrast to the HumanNet-XC, which contains only human-derived functional networks, HumanNet-XI includes interologs derived from five laboratory model organisms (*Caenorhabditis elegans, Drosophila melanogaster, Danio rerio, Mus musculus* and *Saccharomyces cerevisiae*) and four additional vertebrates: *Canis lupus familiaris* (dog), *Bos taurus* (cattle), *Rattus norvegicus* (Rat) and *Gallus gallus* (chicken). HumanNet-XI contains 17 303 genes and 418 525 links.

The fourth level network, HumanNet-XN, is a fully extended functional gene network by both co-citation and interologs. Interologs derived from non-human species provided 101 036 more links to HumanNet-XC, yet its genome coverage only increased from 94.6 to 95.3%. The most comprehensive network, HumanNet-XN, contains 17 929 genes and 525 537 links.

### New types of data used for HumanNet v2

We incorporated functional associations inferred from two new types of data to the updated version of HumanNet. We inferred functional associations from co-annotations by pathway database. If a gene is involved in many different pathways, it may not belong to a specific pathway. Similarly, co-annotation involving such genes would be only weak indication of functional coupling. Thus, we measured the significance of functional association for given co-annotations by Fisher’s exact test, giving more weight on gene pairs that share larger proportion of annotated pathways for each gene. We used pathway annotations by KEGG (22), BioCarta (23) and Recactome (24) databases. Network inference from pathway databases resulted in 125 550 links among 7512 human genes.

Another new type of data used for updating HumanNet was co-essentiality. Recently, several large-scale essential gene screens were conducted across hundreds of cancer cell lines using the shRNA and CRISPR-Cas9 systems. Functionally associated human genes tend to have correlations of essentiality profiles across many cancer cell lines (17). We obtained the functional links inferred from associations between essentiality profiles based on over 100 genome-scale pooled-library shRNA screens and over 400 CRISPR-Cas9 screens from cancer cell lines, which are downloadable from the PICKLES database (25). Network inference from co-essentiality resulted in 71 243 links among 4052 human genes.

### Data source extensions

To improve HumanNet, we also extended the sources of each data type (summarized in Supplemental Table 1). The co-citation network of HumanNet v2 is based on ∼300 000 full-text articles from PubMed Central, whereas ∼750 000 Medline abstracts were used for the co-citation network of the previous version of HumanNet. Sources of PPI data were also substantially extended. The number of database and high-throughput assay sets (Supplemental Table 2) used for human-derived PPI networks increased from 5 to 14 and 3 to 12, respectively. As a result, the number of non-redundant PPIs of HumanNet v2 is 158 499 (connecting 15 352 genes), whereas the PPI network of HumanNet v1 has 60 287 links among 9428 genes. Given that PPIs generally provide high-quality functional associations, this substantially expanded PPI network will significantly improve the generation of functional hypotheses. To update the co-expression networks, we used 125 microarray-based and 33 RNA-seq-based gene expression omnibus (GEO) (26) series (GSEs) (16 220 samples in total) (Supplemental Table 3), whereas only 21 microarray-based GSEs (1603 samples in total) were used in the previous version. Thus, the amount of expression profile data for co-expression analysis has been increased by more than 10-fold. HumanNet includes networks based on genomic context associations (GN and PG). We utilized 1748 prokaryotic (1626 bacterial and 122 archaeal) genomes and 996 metagenomes (754 from human and 242 from ocean) (27,28) to analyze the genomic context associations for HumanNet v2, whereas only 432 prokaryotic (393 bacterial and 31 archaeal) genomes were used for HumanNet v1.

### Network inference algorithm enhancement

Since the release of the first version of HumanNet, we have significantly improved the network inference algorithms for each data type. We found that associations between the phylogenetic profiles of proteins showed a higher correlation with functional association within each domain of life: Archaea, Bacteria, and Eukaryota (29). Thus, for HumanNet v2, we measured the associations between phylogenetic profiles that comprise reference genomes from each domain of life, then integrated the networks based on domain-specific profiles into a single network (PG).

For the previous version of HumanNet, we inferred functional associations by gene neighborhood using only probability-based measures (30). We later found that probability-based and distance-based measures (31) of gene neighborhood are complementary and that their integration could significantly improve network quality (20). Thus, we generated two functional networks using probability- and distance-based measures of gene neighborhood. We also found that distance-based gene neighborhoods across metagenomes correlated with functional associations (32). We could infer two functional networks by gene neighborhood analysis using 754 human microbiomes (27) and 242 ocean metagenomes (28). The final gene neighborhood network (GN) was constructed by integrating the four networks.

The human gene network based on protein domain profiles for HumanNet v2 was improved by using a weighted mutual information (WMI) score that measured the mutual information (MI) between domain profiles of proteins by giving a higher weight to rarer protein domains (19).

### Systematic network evaluation for disease gene discovery

Recently, a systematic network evaluation for their ability to retrieve disease gene sets was conducted for 21 human gene networks, including the previous version of HumanNet (33). The study reported that CPDB (12), GeneMania (13), GIANT (7) and STRING (11) had the best performance in terms of retrieval of literature-curated disease gene sets by DisGeNET (34) and sets of disease candidate genes mapped by *P* < 5e-08 from the GWAS catalog (35). To confirm these results and to evaluate the new human gene networks of HumanNet v2, we evaluated the four best performed gene networks reported by the aforementioned study, another large-scale human functional network, FunCoup (14), and HumanNet v2 for disease gene predictions. Importantly, we used ‘time-stamped benchmarking’ strategy (36) to avoid biased evaluation by co-citation links of HumanNet and STRING. Co-citation links of HumanNet v2 were captured from papers published until 2015. Thus, we used disease-associated genes identified via GWAS published only after 2016 for each trait of the GWAS catalog. With this timestamp filtration, we could obtain 231 traits that contain more than 10 genes mapped by *P* < 5e-08 from the GWAS catalog. Since the latest version of STRING was published in 2016, we expected that the same gene sets could be used for unbiased evaluation of STRING.

We first assessed network accuracy for identifying two genes involved in the same human diseases. We found that two first level networks, HumanNet-PI and HumanNet-CF, to have the worse accuracy than the integrated functional network, HumanNet-FN in terms of connecting gene pairs linked to the same diseases annotated by DisGeNET or GWAS catalog with timestamp filtration as a function of the coverage of the database genes (Figure 1B). This result is consistent with the observation that all of the best performing human gene networks reported by the aforementioned study were functional networks rather than PPI networks (33). We found HumanNet-XC to have the best performance in identifying gene pairs for the same diseases. Notably, incorporating interologs into HumanNet-FN and HumanNet-XC did not notably improve network precision compared with HumanNet-XI and HumanNet-XN, respectively. To evaluate contribution of each evidence to the integrated gene network, accuracy and genome coverage of networks by each data type were also assessed based on the same disease annotations (Supplemental Figure 1).

**Figure 2.**
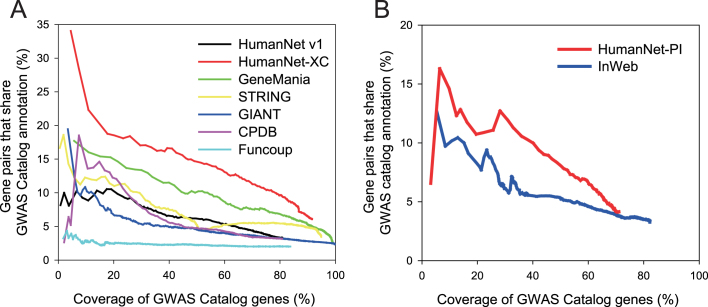
Assessment of human functional gene networks (**A**) and PPI networks (**B**) for genes linked to the same human diseases (defined by GWAS catalog with timestamp filtration) as a function of the coverage of the database genes.

Next, we compared the best performing HumanNet-XC with the previous HumanNet (v1) as well as five other human gene networks, and found that HumanNet-XC outperformed all the other human gene networks (Figure 2A). In addition, we observed that HumanNet-PI has overall higher accuracy than another scored human PPI network, InWeb (Figure 2B). These results indicate that HumanNet v2 might provide the most appropriate networks for disease research by utilizing protein physical interactions as well as functional associations.

**Figure 3.**
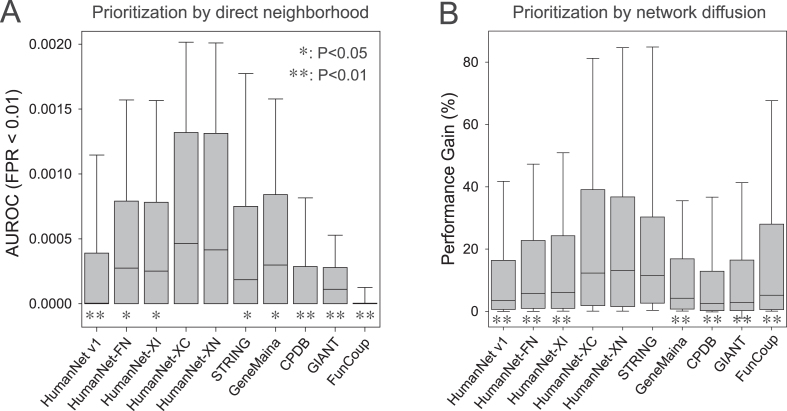
Assessment of predictive ability of networks for unbiased GWAS catalog disease gene sets based on the distribution of (**A**) the area under receiver operating characteristic curve (AUROC) until 1% of false positive rate (FPR < 0.01) and (**B**) performance gain scores based on area under precision recall curve (AUPRC). For each box-and-whisker plot, the boundaries of the box represent the first and third quartiles and the whiskers represent the 10th and 90th percentiles. Significance of performance difference from that of HumanNet-XC is indicated by asterisk (*: *P* < 0.05, **: *P* < 0.01, Wilcoxon rank sum test).

Next, we evaluated the networks for their ability to retrieve disease gene sets. The network performance for disease gene recovery correlates with the efficiency of disease gene discovery by network-based gene prioritization. Network-based gene prioritization for diseases can use two alternative strategies: direct neighborhood and network diffusion (37). Direct neighborhood methods prioritize genes using the disease information of their directly connected network neighbors only (38,39). In contrast, network diffusion methods prioritize genes by propagating disease information throughout the entire network (40). Recently, network diffusion methods have increased in popularity, and the web server of the previous HumanNet version also employed network diffusion for disease gene prioritization. However, more recently, multiple studies have shown that direct neighborhood is generally more efficient than network diffusion in obtaining disease genes in the top predictions (41,42). Because typically only a few hundred candidates at most are considered for the follow-up functional analysis, we benchmarked the retrieval efficiency of disease genes by the area under the receiver operating characteristic curve (AUROC) until a false positive rate of 1% (FPR < 0.01). With this benchmarking analysis, we found HumanNet-XC and HumanNet-XN to have significantly better performance than all other networks by direct neighborhood with the unbiased disease gene sets (Figure 3A). We observed similar results for AUROC until FPR of 2% and 5% (Supplemental Figure 2). In consistent with the results of previous systematic evaluation, HumanNet v1 showed worse performance than STRING, GeneMania, and GIANT with the time-stamped benchmarking, indicating large influence of co-citation information on the earlier version of HumanNet (33).

It is also possible to prioritize disease genes with network diffusion techniques such as random walk with the restart model (40). For benchmarking the retrieval efficiency of disease gene sets by network diffusion, we used ‘performance gain’ scores based on the area under the precision recall curve (AUPRC) as described in a previous study on systematic network evaluations (33). With this benchmarking analysis, we found HumanNet-XC, HumanNet-XN, and STRING to have significantly better performance than other networks (Figure 3B).

Notably, in all of the above benchmarking analysis, we did not observe a significant increase in performance by incorporating interologs (*P* > 0.05 for HumanNet-FN versus HumanNet-XI and for HumanNet-XC versus HumanNet-XN, Wilcoxon rank sum test). These results suggest that many of the evolutionarily conserved gene links for the same diseases are now captured directly in human-derived data. However, we cannot exclude the possibility that intrologs can improve gene prioritization for non-pathogenic cellular processes such as core metabolic pathways.

## WEB INTERFACE IMPROVEMENT

### Implementation of a new user interface

We implemented back-end and front-end servers for HumanNet v2 to facilitate effective interactions with users. For the back-end server implementation, we used Redis (https://redis.io), an in-memory DB which reduces the data loading time significantly compared with that from a hard drive. We designed the back-end interface as an Application Programming Interface (API) to communicate with the front-end server and also job requests from users. We employed several open-sourced Cascading Style Sheet components and JavaScript libraries for front-end server implementation. We designed the website layout using Bootstrap4 and its components (https://getbootstrap.com). Cytoscape.js (43) and its extensions, ‘cytoscape.js-cose-bilkent’ (from https://doi.org/10.5281/zenodo.1098231) and ‘cytoscape.js-panzoom’ (from http://doi.org/10.5281/zenodo.835037) were employed to provide the graph and network visualization.

### Disease-focused hypothesis generation

The HumanNet v2 web server facilitates human disease research by predicting disease genes or disease annotations. Network-based disease gene predictions are generally based on the network connections to the genes known to be involved in the disease. We dubbed these known disease genes ‘guide genes’ because they guide the network-based predictions of new disease gene candidates. We can estimate the predictive performance of networks based on the efficiency of guide gene recovery. The HumanNet v2 server uses a direct neighborhood approach rather than network diffusion for network-based gene prioritization, because at most a few hundred candidates are considered for follow-up functional analysis and direct neighborhood generally outperforms network diffusion methods for the early retrieval of guide genes (41,42). The HumanNet v2 server uses HumanNet-XC as a default network, because it showed the best performance for disease gene recovery in our benchmarking analyses.

**Figure 4.**
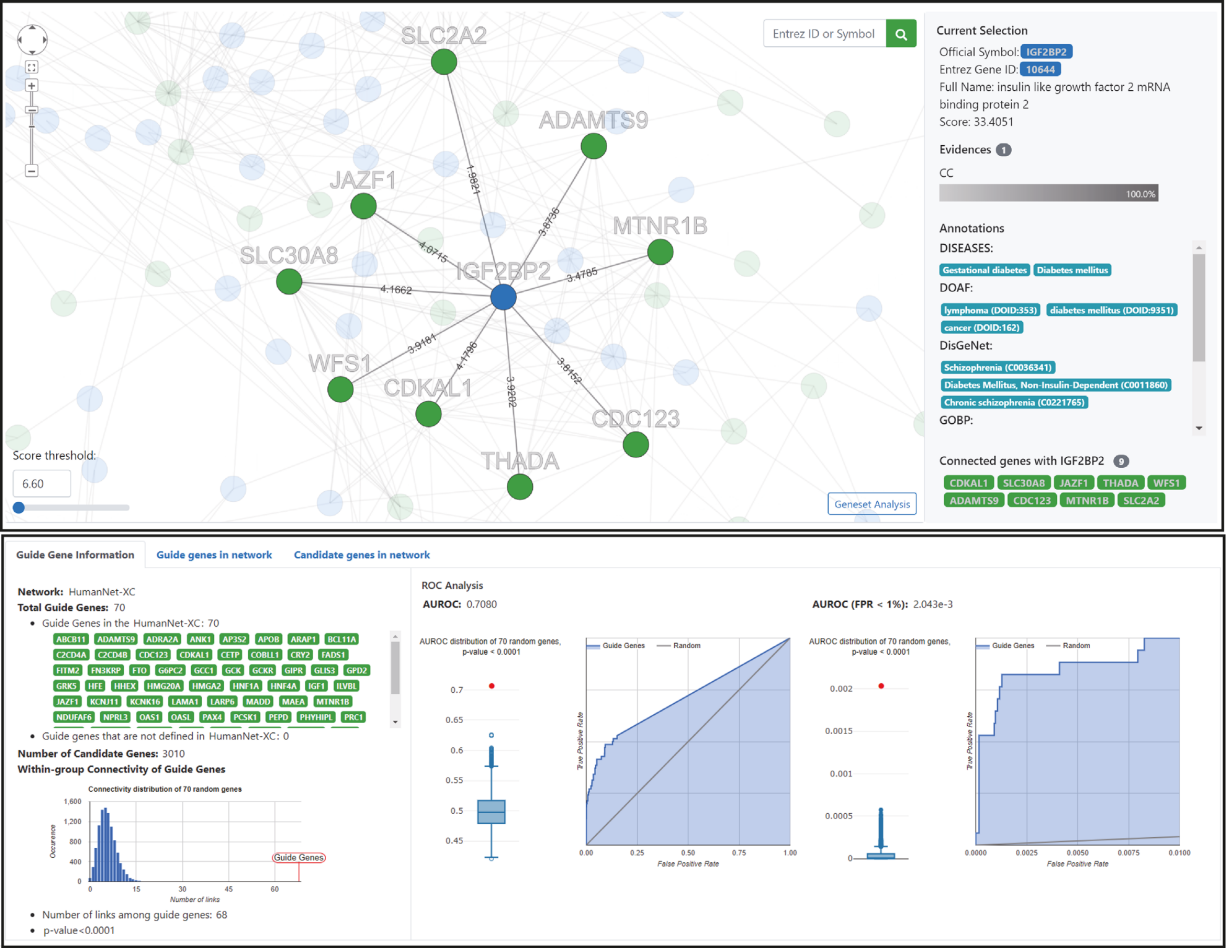
Screenshots of the HumanNet reports page for the network-based disease gene prediction using HumanNet-XC based on submission of 70 genes for type 2 diabetes mellitus (defined by DISEASES) as guide (query) genes. The upper panel shows the interactive network viewer, visualizing a network of guide genes (green nodes) and their top 100 direct neighbors, which can be interpreted as putative candidate genes (blue nodes). Here, the local subnetwork of the third ranked candidate, IGF2BP2 and its neighbors is highlighted. The retrieved gene IGF2BP2 is already annotated for diabetes mellitus by DISEASES, DOAF and DisGeNET, serving to validate the specific prediction result. The lower panel reports data on the guide genes, including the statistical significance of within group connectivity of guide genes, and the observed network performance for guide gene recovery reported as ROC curves.

Using multiple guide genes for network-based predictions is desirable, because predictions based on multiple network connections are more confident due to the ensemble effect. The functional coherence of guide genes would be a meaningful indicator of their effectiveness. Therefore, the HumanNet server reports on the significance of within-group connectivity of guide genes using 10 000 random gene sets of the same size (Figure 4, lower panel). The HumanNet server also reports on ROC plots, which indicate the predictive performance of networks for a disease based on the efficiency of guide gene recovery. To evaluate statistical significance of the observed AUROC score, HumanNet v2 server generates null models using 10 000 random gene sets of the same size. Users can submit pre-defined disease gene sets from DisGeNET (34), DISEASES (44), Disease Ontology Annotation Framework (DOAF) (45), GWAS catalog (35) and Human Phenotype Ontology (HPO) (46). Users can also submit a set of genes targeted by a drug based on DiSigDB (47). Thus, predictions guided by the DiSigDB gene set are likely candidates of novel targets for the same drug. HumanNet users can also predict disease annotations of a gene based on the network-neighbors. For a query gene, the HumanNet server collects disease annotations from its network neighbors and lists them starting from the most enriched one.

### Interactive network viewer

Network-based disease gene prediction generates a network of guide genes and new candidate genes for disease. Further investigation of the disease gene network would provide functional insights which might be useful for narrowing down final candidates and for mode-of-action studies. Therefore, we designed a network viewer enabling users to conduct interactive analyses of the disease gene network. The HumanNet v2 server generates a network of guide genes and the top 100 candidate genes for the disease. Initially, the entire network appears in the viewer to give a brief idea of the disease gene network, but soon after, all the candidate genes disappear. Then, users can select different numbers of top candidate genes for a new disease network by thresholding the prediction score (Figure 4, upper panel). Users can select a particular gene of the disease network not only from the network viewer but also from the table of candidate genes. The network viewer highlights a local subnetwork of the choice of gene and its network neighbors. Users can also see additional information such as annotations of the GO biological process and diseases for the chosen gene and supporting evidence for the local network connections. The interactive thresholding for candidate gene selection allows users to consider various disease gene networks with different trade-offs between degree of confidence and coverage. Disease-association for the selected group of candidate genes can be summarized by gene-set analysis (GSA). Users can select the top *N* candidate genes and run GSA with not only GO biological processes but also annotated disease genes from DisGeNET (34), DISEASES (44), DOAF (45) and HOP (46).

## CONCLUSION

In this report, we present an updated HumanNet by incorporating new types of data, extending data sources and improving network inference algorithms. The new HumanNet was designed to have an inclusive, four level hierarchy of human gene networks. Based on our benchmarking results for their performance of disease gene recovery, we conclude that HumanNet serves as one of the better human gene networks for prioritizing disease-linked genes and reconstructing disease-relevant gene modules. We recommend HumanNet-XC for most network-based disease research, but other networks will be useful for other purposes. For example, HumanNet-PI is recommended for the mode-of-action studies of disease mutation, HumanNet-FN for more conservative predictions of disease genes and HumanNet-XN for studies requiring the most comprehensive networks. Due to the continuous growth of omics data repositories and the advent of new types of functional genomics data such as single cell transcriptome profiles, we might be able to keep improving HumanNet in the future. With a highly interactive web server for generating hypotheses, we expect HumanNet to be a highly useful *in silico* resource for the study of human diseases.

## Supplementary Material

Supplementary Data
