# Supplementary material for: HumanNet v2: human gene networks for disease research
Source: Nucleic Acids Res. 2018 Nov 10;47(Database issue):D573–80. doi: 10.1093/nar/gky1126 (PMC6323914; doi:10.1093/nar/gky1126)
Supplement: Supplementary Data [file gky1126_supplemental_files.zip › HumanNetv2 Supplemental online information Nov.02.2018.pdf]

Supplemental Online Information

HumanNet v2: human gene networks for disease research

Sohyun Hwang, Chan Young Kim, Sunmo Yang, Eiru Kim, Traver Hart, Edward M. Marcotte, and Insuk Lee

Supplemental Methods

Genome, function, disease, and drug target annotations for *Homo sapiens*.

We consider 18,805 protein-coding genes of *H. sapiens* downloaded from Consensus CDS (CCDS) Database release 18 (1) for network modeling and analysis. We compiled seven databases of function, disease, and drug target annotations for the analysis of human genes: Gene Ontology biological process (GOBP) annotations (http://www.geneontology.org as of April 4, 2018) (2), curated annotations of DisGeNET (http://www.DisGeNET.org as of June 8, 2018) (3), Disease Ontology Annotation Framework (DOAF) annotations (http://doa.nubic.northwestern.edu/ as of September 3, 2016) (4), frequent-featured annotations of Human Phenotype Ontology (HPO) (https://hpo.jax.org/ as of March 9, 2018) (5), disease gene annotations with more than 3-star scores of DISEASES (https://diseases.jensenlab.org) (6), disease-genes associations mapped by *P*<5e-08 from GWAS catalog (https://www.ebi.ac.uk/gwas/ as of September 14, 2018) (7), and drug-target annotations of DSigDB Release 1 (http://tanlab.ucdenver.edu/DSigDB/DSigDBv1.0/) (8).

Benchmarking and Bayesian integration of co-functional links

We constructed a co-functional network of human genes using a supervised machine learning approach. To train a functional gene network, we compiled positive gold standard gene pairs from GOBP (2) and MetaCyc (9) metabolic process annotations by pairing genes that share annotation terms, which indicate that two genes belong to the same biological or metabolic process terms. We excluded 9 GOBP terms annotating more than 90 genes and superpathway terms of MetaCyc to reduce functional bias in the benchmark set. Negative gold standard gene pairs were intrinsically generated by pairing two genes that were annotated by GOBP or

MetaCyc but which do not share any annotation terms. The total number of positive and negative gold standard gene pairs was 124,411 and 13,273,665, respectively, between 5,177 genes.

The likelihood of a co-functional link supported by the given data was measured using a Bayesian statistic framework (10). The log likelihood score (*LLS*) of co-functional links between genes was calculated by the following equation:

$$LLS = \ln\left(\frac{P(L|E)/P(\neg L|E)}{P(L)/P(\neg L)}\right),$$

where $P(L|E)$ and $P(\neg L|E)$ are the probability of positive and negative gold standard links supported by the given data, respectively. $P(L)$ and $P(\neg L)$ are the probability of positive and negative gold standard links, respectively. To compute *LLS* of gene pairs ordered by evidence-derived score, we used bins of 1000 gene pairs, in which the number of annotated human gene pairs by gold standard data usually resides in the range between 50 and 300.

For the co-functional links supported by multiple types of data, as well as multiple *LLS* values, we integrated the scores using the weighted sum (*WS*) method as described in (10):

$$WS = L_0 + \sum_{i=1}^{n} \frac{L_i}{D \cdot i}, \ for\ all\ L \geq T,$$

where L_0 is the maximum *LLS* for the given link and i is the rank index for the rest of *LLS* values by decreasing order. *D* is a free parameter of weight factor and *T* is a minimum score threshold to be considered for the integration. We optimized the choice of the weighting parameters *D* and *T* using recall-precision analysis by maximizing the area under a plot of *LLS* versus the number of genes participating in the linkages.

Summary of updates for HumanNet v2

HumanNet was updated using three strategies: including new types of data (co-essentiality and pathway database), extending sources for each data type (using more reference genomes for genome context methods, more expression data for co-expression analysis, more databases and high throughput assay sets for PPI networks, more research articles for co-citation analysis), and improving network inference algorithms (for association of protein domain profiles and genome context methods). These update for HumanNet v2 construction are summarized in **Supplemental Table 1**.

Co-functional links based on protein-protein interactions derived from high-throughput experiments (HT) and literature curation (LC)

Co-functional links can be supported by binary protein-protein interactions (PPI) and protein complexes mapped by experimental techniques such as yeast-two-hybrid (Y2H) assay and affinity purifications. We compiled PPIs from high throughput experiments: seven protein complex mapping datasets (11-17) and five binary PPI screen datasets (18-22) (**Supplemental Table 2**). For the binary interaction datasets, we applied Fisher's exact test to rank the order of PPIs. If there are multiple links for the same gene pairs due to the existence of protein isoforms, we selected a single gene pair with the highest score based on the PPI data.

PPIs have been mapped by small and medium-scale assays and are generally reported as individual research articles. There exist many databases for PPIs collected from research articles by literature curation. We obtained PPIs by literature curation from a single meta-database, IRefIndex version 14 (23) which consolidated PPIs from many original databases. IRefIndex contains PPIs not only from small- and medium-scale studies but also from high throughput screens. To exclude PPIs based on high throughput screens, we filtered out PPIs derived from the 12 articles used for the HT network. In addition, we excluded PPIs by orthology-based transfer from other organisms, interologs (24). We also ranked the PPIs by significance of interactions using Fisher's exact test. *LLS* values were assigned to each gene pairs based on the regression between –log(*P*) and *LLS* for bins of 1000 gene pairs with the order of decreasing –log(*P*).

Co-functional links inferred from co-citation of human genes (CC)

Two genes that are functionally associated tend to be co-cited across research articles. Therefore, co-functional links were identified based on statistical significance of co-citation of two genes across PubMed abstracts (25). However, there are functionally associated genes that are co-cited in the main text rather than the abstract of the article. To increase the sensitivity of the co-citation approach, we used full-text research articles with the Medical Subject Headings (MeSH) term of “humans” in PubMed Central (PMC, http://www.ncbi.nlm.nih.gov/pmc/) abstracts and scanned for any human gene name in the HUGO Gene Nomenclature database in those full-texts (26). We found a total of 293,139 articles containing human gene names, and

then calculated the significance of functional association for the given co-citation of two genes across the articles by one-tail Fisher's exact test, giving more weight on gene pairs share larger propotion of cited papers for each gene. Finally, we assigned *LLS* to each gene pairs based on regression model between –log(*P*) and *LLS* for bins of 1000 gene pairs with the order of decreasing –log(*P*).

Co-functional links inferred from co-expression of genes (CX)

Functionally associated genes tend to co-express across diverse experimental conditions. Thus, we can infer co-functional links based on significant patterns of co-expression (27). We measured the significance of correlation of expression between two genes across samples using the Pearson correlation coefficient (*PCC*). To construct co-expression networks of human genes, we exploited a large depository of transcriptome data, Gene Expression Omnibus (GEO) (28). After testing 2,060 GEO series (1,919 GSEs for microarray data sets and 141 GSEs for RNA-seq data sets with more than 12 samples), we selected 158 GSEs (125 microarray-based GSEs and 33 RNA-seq-based GSEs) from which we observed a strong correlation between the *PCC* scores and *LLS* values for bins of 1000 gene pairs with the order of decreasing *PCC* (**Supplemental Table 3**). We assigned *LLS* to each gene pair based on the regression model between *PCC* and *LLS*. Then, we integrated the 158 co-expression networks into a single network using the weighted sum method described above.

Co-functional links inferred from associations between protein domain profiles (DP)

Domains are recurring functional motifs of proteins. Because domains are the structural, functional, and evolutionary units of proteins, proteins that share a similar set of domains are likely to be functionally associated (29). We first constructed the domain occurrence profiles of proteins based on the InterPro database (30). We previously reported that the measure of association between domain profiles with a weighted mutual information (WMI) score, which accounts greater functional importance for rarer domains, generally results in a stronger correlation with likelihood of functional association (31). We thus used WMI to infer co-functional links based on the associations between the protein domain profiles of human genes.

Co-functional links inferred from genomic contexts (PG and GN)

We used two different types of genomic context association between human proteins to infer co-functional links between genes: phylogenetic associations and gene neighborhood. The association between the phylogenetic profiles of the two genes reflects the degree of their co-inheritance during speciation due to the functional constraints (32). We first ran BLASTP (33) to identify orthologs of all human proteins in fully sequenced 1,626 bacteria, 122 archaea, and 396 eukaryote genomes. The phylogenetic profiles for all human proteins were constructed based on the blast-hit scores, and the association between the two profiles was measured by the mutual information (MI) scores (32). We previously found that the association of phylogenetic profiles based on each domain of life (Archaea, Bacteria, Eukarya) showed a stronger correlation with the likelihood of functional association between genes (34). We observed a strong positive correlation between the MI score and the likelihood of functional links with 1,626 bacterial genomes and 122 archaeal genomes, individually. We constructed two co-functional networks based on the two domain-specific phylogenetic profiles and integrated them into a single network using the weighted-sum method.

Bacterial genes located in chromosomal proximity generally work together for the same biological processes. For example, genes that belong to a single bacterial operon are transcribed as a single transcript and co-translated to produce multiple proteins to conduct a metabolic process simultaneously. Therefore, if two bacterial genes are in chromosomal neighborhood across many genomes, the two genes are likely to have a functional association (35). The concept of gene neighborhood is not merely applied to bacterial genes. We can also infer co-functional links according to the proximity of bacterial orthologs of human genes across bacterial genomes. There are two different measures of gene neighborhood: distance- and probability-based gene neighborhood (36). We previously found that these two measures of gene neighborhood are complementary, thus we can construct a more comprehensive and accurate network by integrating them. Therefore, we generated two networks based on the gene neighborhood methods using 1,746 fully sequenced bacterial genomes. More recently, we found that we could infer co-functional links by a distance-based gene neighborhood with metagenomes (37). We constructed a co-functional network based on metagenomic assembly contigs derived from 754 humans (38) and 242 ocean samples (39). The final network for the gene neighborhood is based on the integration of all above four networks (two based on fully sequence bacterial genomes and two based on human and ocean metagenomes) using the

weighted-sum method.

Co-functional links based on interologs from five other species (IL)

PPIs are often evolutionarily conserved in different species and are called interologs (24). We first compiled human interologs from the PPIs of previously constructed functional gene networks for five laboratory model organisms: *Caenorhabditis elegans* (40), *Drosophila melanogaster* (41), *Danio rerio* (42), *Mus musculus* (43), and *Saccharomyces cerevisiae* (44). They are five literature-curated PPIs in the five species, three high-throughput PPIs in *C. elegans*, *D. melanogaster*, and *S. cerevisiae*. We also compiled inferred PPI networks based on genetic interaction profile associations and the three-dimensional structure of proteins of *S. cerevisiae*. We collected additional PPIs from iRefIndex database (23) for four vertebrate species: *Canis lupus familiaris* (dog), *Bos taurus* (cattle), *Rattus norvegicus* (Rat), and *Gallus gallus* (chicken). To identify orthologs between human proteins and those of nine other species, we used the Inparanoid algorithm (45). The *LLS* scores of the original gene pairs were reweighted using the Inparalog score (*IWLLS*). We assigned new *LLS* values to each gene pair based on the correlation between *IWLLS* and *LLS* for bins of 1000 gene pairs with the order of decreasing *IWLLS*. These ten networks of human genes by orthology-based transfer were then integrated into a single network by the weighted sum method.

Co-functional links inferred from co-annotation by pathway databases (DB)

Our current knowledge provides pathway annotations for many human genes. If two genes are significantly more co-annotated by pathway terms than by random chance, their functional association might be more strong or specific. We measured the significance of functional association for given co-annotations by Fisher's exact test, giving more weight on gene pairs that share larger proportion of annotated pathways for each genes. We then assigned *LLS* to each gene pair based on the correlation between –log(*P*) and *LLS* scores for bins of 1000 gene pairs with the order of decreasing –log(*P*). We constructed functional networks by exploiting three pathway databases: KEGG as of January 5, 2017 (https://www.genome.jp/kegg/) (46), BioCarta as of January 5, 2017 (https://cgap.nci.nih.gov/Info/CGAPDownload) (47), and Reactome as of January 3, 2017 (https://reactome.org/) (48). For KEGG pathways, we excluded the pathways that annotated more than 300 genes. For Reactome pathways, we used

pathways supported by GO evidence code of TAS (Traceable Author Statement) only. We conducted co-annotation analysis for each of the three databases to construct three networks, then integrated them into a single network by the weighted-sum method.

Co-functional links inferred from co-essentiality (CE)

Recently several large-scale essential gene screens were conducted across many cancer cell lines using the shRNA and CRISPR-Cas9 systems. Functionally associated human genes often show similar essentiality profiles across many cancer cell lines. We inferred co-functional links from co-essentiality analyses based on over 100 high-quality and genome-scale shRNA-based essentiality profiles and over 400 CRISPR-based essentiality profiles, and the resultant networks are available at https://doi.org/10.1101/134346 and https://doi.org/10.1101/328880. We assigned an *LLS* score to each gene pair based on the correlation between the original edge scores and *LLS* for bins of 1000 gene pairs in decreasing order. The two co-essentiality networks were then integrated into a single network by the weighted sum method.

Network data processing

Since we considered 18,805 protein-coding genes of *H. sapiens* by CCDS Database for HumanNet construction, we also kept only interactions where both interactors were one of the 18,805 genes for all other human gene networks in this study. We then removed self- and redundant-interactions from each of the networks. We used full size network obtained from the original databases except GIANT which provides more than 38 million interactions. For GIANT, we used only interactions with confidence probability > 0.1.

Network evaluation for ability to retrieve disease gene sets

We used two types of disease gene sets: literature-curated disease gene sets and disease candidate gene sets derived from GWAS. We compiled 2,297 literature-curated disease gene sets with more than 10 member genes from DisGeNET (3). For the candidate disease genes, we compiled genes mapped by $P < 5e\text{-}08$ from the GWAS catalog (https://www.ebi.ac.uk/gwas/ as of September 14, 2018) (7). To evaluate network performance of disease gene predictions with no bias toward co-citation information of HumanNet and STRING, we used time-stamped benchmarking approach (49). HumanNet contains co-citation links based on papers published

until 2015. Therefore, we used disease gene candidates identified by GWAS only after 2016. By taking traits annotating at least 10 genes, we obtained 231 disease gene sets for the unbiased benchmarking. Since the latest version of STRING was published in 2016, we expect that the same benchmarking gene sets could be also used for evaluation of STRING.

The retrieval of true disease genes within the top predictions is important because at most only a few hundred top candidates are generally carried into the follow-up functional analysis. Therefore, we measured the area under the receiver operating characteristic curve (AUROC) to a 1% false positive rate (FPR < 0.01). We also benchmarked using the area under the precision-recall curve (AUPRC) as described in a previous work (50). Briefly, we generated 50 null networks for each network by shuffling individual edges while preserving node degrees. We repeated random split of each gene set into two groups 50 times. Therefore, we performed 2500 tests with different combinations between 50 random split of gene sets and 50 randomized networks. We then predicted a group of genes using another group of genes with random-walk with restart method and measured the AUPRC. For each network, the performance gain was calculated by following equation:

$$\frac{AUPRC\ of\ given\ network - median\ AUPRC\ of\ its\ null\ networks}{median\ AUPRC\ of\ its\ null\ networks} \times 100\ (\%).$$

Supplemental References

1. Pujar, S., O'Leary, N.A., Farrell, C.M., Loveland, J.E., Mudge, J.M., Wallin, C., Giron, C.G., Diekhans, M., Barnes, I., Bennett, R. *et al.* (2018) Consensus coding sequence (CCDS) database: a standardized set of human and mouse protein-coding regions supported by expert curation. *Nucleic acids research*, **46**, D221-D228.

2. Ashburner, M., Ball, C.A., Blake, J.A., Botstein, D., Butler, H., Cherry, J.M., Davis, A.P., Dolinski, K., Dwight, S.S., Eppig, J.T. *et al.* (2000) Gene ontology: tool for the unification of biology. The Gene Ontology Consortium. *Nature genetics*, **25**, 25-29.

3. Pinero, J., Bravo, A., Queralt-Rosinach, N., Gutierrez-Sacristan, A., Deu-Pons, J., Centeno, E., Garcia-Garcia, J., Sanz, F. and Furlong, L.I. (2017) DisGeNET: a comprehensive platform integrating information on human disease-associated genes and variants. *Nucleic acids research*, **45**, D833-D839.

4. Xu, W., Wang, H., Cheng, W., Fu, D., Xia, T., Kibbe, W.A. and Lin, S.M. (2012) A framework for annotating human genome in disease context. *PloS one*, **7**, e49686.

5. Kohler, S., Vasilevsky, N.A., Engelstad, M., Foster, E., McMurry, J., Ayme, S., Baynam, G., Bello, S.M., Boerkoel, C.F., Boycott, K.M. *et al.* (2017) The Human Phenotype Ontology in 2017. *Nucleic acids research*, **45**, D865-D876.

6. Pletscher-Frankild, S., Palleja, A., Tsafou, K., Binder, J.X. and Jensen, L.J. (2015) DISEASES: text mining and data integration of disease-gene associations. *Methods*, **74**, 83-89.

7. MacArthur, J., Bowler, E., Cerezo, M., Gil, L., Hall, P., Hastings, E., Junkins, H., McMahon, A., Milano, A., Morales, J. *et al.* (2017) The new NHGRI-EBI Catalog of published genome-wide association studies (GWAS Catalog). *Nucleic acids research*, **45**, D896-D901.

8. Yoo, M., Shin, J., Kim, J., Ryall, K.A., Lee, K., Lee, S., Jeon, M., Kang, J. and Tan, A.C. (2015) DSigDB: drug signatures database for gene set analysis. *Bioinformatics*, **31**, 3069-3071.

9. Caspi, R., Billington, R., Fulcher, C.A., Keseler, I.M., Kothari, A., Krummenacker, M., Latendresse, M., Midford, P.E., Ong, Q., Ong, W.K. *et al.* (2018) The MetaCyc database of metabolic pathways and enzymes. *Nucleic acids research*, **46**, D633-D639.

10. Lee, I., Date, S.V., Adai, A.T. and Marcotte, E.M. (2004) A probabilistic functional network of yeast genes. *Science*, **306**, 1555-1558.

11. Wan, C., Borgeson, B., Phanse, S., Tu, F., Drew, K., Clark, G., Xiong, X., Kagan, O., Kwan, J., Bezginov, A. *et al.* (2015) Panorama of ancient metazoan macromolecular complexes. *Nature*, **525**, 339-344.

12. Sowa, M.E., Bennett, E.J., Gygi, S.P. and Harper, J.W. (2009) Defining the human deubiquitinating enzyme interaction landscape. *Cell*, **138**, 389-403.

13. Hutchins, J.R., Toyoda, Y., Hegemann, B., Poser, I., Heriche, J.K., Sykora, M.M., Augsburg, M., Hudecz, O., Buschhorn, B.A., Bulkescher, J. *et al.* (2010) Systematic analysis of human protein complexes identifies chromosome segregation proteins. *Science*, **328**, 593-599.

14. Ewing, R.M., Chu, P., Elisma, F., Li, H., Taylor, P., Climie, S., McBroom-Cerajewski, L., Robinson, M.D., O'Connor, L., Li, M. *et al.* (2007) Large-scale mapping of human protein-protein interactions by mass spectrometry. *Mol Syst Biol*, **3**, 89.

15. Marcon, E., Ni, Z., Pu, S., Turinsky, A.L., Trimble, S.S., Olsen, J.B., Silverman-Gavrila, R., Silverman-Gavrila, L., Phanse, S., Guo, H. *et al.* (2014) Human-

chromatin-related protein interactions identify a demethylase complex required for chromosome segregation. *Cell reports*, **8**, 297-310.

16. Hein, M.Y., Hubner, N.C., Poser, I., Cox, J., Nagaraj, N., Toyoda, Y., Gak, I.A., Weisswange, I., Mansfeld, J., Buchholz, F. *et al.* (2015) A human interactome in three quantitative dimensions organized by stoichiometries and abundances. *Cell*, **163**, 712-723.

17. Huttlin, E.L., Bruckner, R.J., Paulo, J.A., Cannon, J.R., Ting, L., Baltier, K., Colby, G., Gebreab, F., Gygi, M.P., Parzen, H. *et al.* (2017) Architecture of the human interactome defines protein communities and disease networks. *Nature*, **545**, 505-509.

18. Rolland, T., Tasan, M., Charloteaux, B., Pevzner, S.J., Zhong, Q., Sahni, N., Yi, S., Lemmens, I., Fontanillo, C., Mosca, R. *et al.* (2014) A proteome-scale map of the human interactome network. *Cell*, **159**, 1212-1226.

19. Yu, H., Tardivo, L., Tam, S., Weiner, E., Gebreab, F., Fan, C., Svrzikapa, N., Hirozane-Kishikawa, T., Rietman, E., Yang, X. *et al.* (2011) Next-generation sequencing to generate interactome datasets. *Nature methods*, **8**, 478-480.

20. Rual, J.F., Venkatesan, K., Hao, T., Hirozane-Kishikawa, T., Dricot, A., Li, N., Berriz, G.F., Gibbons, F.D., Dreze, M., Ayivi-Guedehoussou, N. *et al.* (2005) Towards a proteome-scale map of the human protein-protein interaction network. *Nature*, **437**, 1173-1178.

21. Wang, J., Huo, K., Ma, L., Tang, L., Li, D., Huang, X., Yuan, Y., Li, C., Wang, W., Guan, W. *et al.* (2011) Toward an understanding of the protein interaction network of the human liver. *Mol Syst Biol*, **7**, 536.

22. Venkatesan, K., Rual, J.F., Vazquez, A., Stelzl, U., Lemmens, I., Hirozane-Kishikawa, T., Hao, T., Zenkner, M., Xin, X., Goh, K.I. *et al.* (2009) An empirical framework for binary interactome mapping. *Nature methods*, **6**, 83-90.

23. Razick, S., Magklaras, G. and Donaldson, I.M. (2008) iRefIndex: a consolidated protein interaction database with provenance. *BMC Bioinformatics*, **9**, 405.

24. Yu, H., Luscombe, N.M., Lu, H.X., Zhu, X., Xia, Y., Han, J.D., Bertin, N., Chung, S., Vidal, M. and Gerstein, M. (2004) Annotation transfer between genomes: protein-protein interologs and protein-DNA regulogs. *Genome research*, **14**, 1107-1118.

25. Stapley, B.J. and Benoit, G. (2000) Biobibliometrics: information retrieval and visualization from co-occurrences of gene names in Medline abstracts. *Pac Symp Biocomput*, 529-540.

26. Yates, B., Braschi, B., Gray, K.A., Seal, R.L., Tweedie, S. and Bruford, E.A. (2017) Genenames.org: the HGNC and VGNC resources in 2017. *Nucleic acids research*, **45**, D619-D625.

27. Yang, S., Kim, C.Y., Hwang, S., Kim, E., Kim, H., Shim, H. and Lee, I. (2017) COEXPEDIA: exploring biomedical hypotheses via co-expressions associated with medical subject headings (MeSH). *Nucleic acids research*, **45**, D389-D396.

28. Barrett, T., Wilhite, S.E., Ledoux, P., Evangelista, C., Kim, I.F., Tomashevsky, M., Marshall, K.A., Phillippy, K.H., Sherman, P.M., Holko, M. *et al.* (2013) NCBI GEO: archive for functional genomics data sets--update. *Nucleic acids research*, **41**, D991-995.

29. Reimand, J., Hui, S., Jain, S., Law, B. and Bader, G.D. (2012) Domain-mediated protein interaction prediction: From genome to network. *FEBS Lett*, **586**, 2751-2763.

30. Finn, R.D., Attwood, T.K., Babbitt, P.C., Bateman, A., Bork, P., Bridge, A.J., Chang, H.Y., Dosztanyi, Z., El-Gebali, S., Fraser, M. *et al.* (2017) InterPro in 2017-beyond protein family and domain annotations. *Nucleic acids research*, **45**, D190-D199.

31. Shim, J.E. and Lee, I. (2016) Weighted mutual information analysis substantially improves domain-based functional network models. *Bioinformatics*, **32**, 2824-2830.

32. Shin, J. and Lee, I. (2017) Construction of Functional Gene Networks Using Phylogenetic Profiles. *Methods in molecular biology*, **1526**, 87-98.

33. Altschul, S.F., Gish, W., Miller, W., Myers, E.W. and Lipman, D.J. (1990) Basic local alignment search tool. *J Mol Biol*, **215**, 403-410.

34. Shin, J. and Lee, I. (2015) Co-Inheritance Analysis within the Domains of Life Substantially Improves Network Inference by Phylogenetic Profiling. *PloS one*, **10**, e0139006.

35. Dandekar, T., Snel, B., Huynen, M. and Bork, P. (1998) Conservation of gene order: a fingerprint of proteins that physically interact. *Trends Biochem Sci*, **23**, 324-328.

36. Shin, J., Lee, T., Kim, H. and Lee, I. (2014) Complementarity between distance- and probability-based methods of gene neighbourhood identification for pathway reconstruction. *Mol Biosyst*, **10**, 24-29.

37. Kim, C.Y. and Lee, I. (2017) Functional gene networks based on the gene neighborhood in metagenomes. *Animal Cells and Systems*, **21**, 301-306.

38. Human Microbiome Project, C. (2012) Structure, function and diversity of the healthy human microbiome. *Nature*, **486**, 207-214.

39. Sunagawa, S., Coelho, L.P., Chaffron, S., Kultima, J.R., Labadie, K., Salazar, G., Djahanschiri, B., Zeller, G., Mende, D.R., Alberti, A. *et al.* (2015) Ocean plankton. Structure and function of the global ocean microbiome. *Science*, **348**, 1261359.

40. Cho, A., Shin, J., Hwang, S., Kim, C., Shim, H., Kim, H., Kim, H. and Lee, I. (2014) WormNet v3: a network-assisted hypothesis-generating server for Caenorhabditis elegans. *Nucleic acids research*, **42**, W76-82.

41. Shin, J., Yang, S., Kim, E., Kim, C.Y., Shim, H., Cho, A., Kim, H., Hwang, S., Shim, J.E. and Lee, I. (2015) FlyNet: a versatile network prioritization server for the Drosophila community. *Nucleic acids research*, **43**, W91-97.

42. Shim, H., Kim, J.H., Kim, C.Y., Hwang, S., Kim, H., Yang, S., Lee, J.E. and Lee, I. (2016) Function-driven discovery of disease genes in zebrafish using an integrated genomics big data resource. *Nucleic acids research*, **44**, 9611-9623.

43. Kim, E., Hwang, S., Kim, H., Shim, H., Kang, B., Yang, S., Shim, J.H., Shin, S.Y., Marcotte, E.M. and Lee, I. (2016) MouseNet v2: a database of gene networks for studying the laboratory mouse and eight other model vertebrates. *Nucleic acids research*, **44**, D848-854.

44. Kim, H., Shin, J., Kim, E., Kim, H., Hwang, S., Shim, J.E. and Lee, I. (2014) YeastNet v3: a public database of data-specific and integrated functional gene networks for Saccharomyces cerevisiae. *Nucleic acids research*, **42**, D731-736.

45. O'Brien, K.P., Remm, M. and Sonnhammer, E.L. (2005) Inparanoid: a comprehensive database of eukaryotic orthologs. *Nucleic acids research*, **33**, D476-480.

46. Kanehisa, M., Furumichi, M., Tanabe, M., Sato, Y. and Morishima, K. (2017) KEGG: new perspectives on genomes, pathways, diseases and drugs. *Nucleic acids research*, **45**, D353-D361.

47. Nishimura, D. (2001) BioCarta. *Biotech Software & Internet Report*, **2**, 117-120.

48. Fabregat, A., Jupe, S., Matthews, L., Sidiropoulos, K., Gillespie, M., Garapati, P., Haw, R., Jassal, B., Korninger, F., May, B. *et al.* (2018) The Reactome Pathway Knowledgebase. *Nucleic acids research*, **46**, D649-D655.

49. Bornigen, D., Tranchevent, L.C., Bonachela-Capdevila, F., Devriendt, K., De Moor, B., De Causmaecker, P. and Moreau, Y. (2012) An unbiased evaluation of gene

prioritization tools. *Bioinformatics*, **28**, 3081-3088.

50. Huang, J.K., Carlin, D.E., Yu, M.K., Zhang, W., Kreisberg, J.F., Tamayo, P. and Ideker, T. (2018) Systematic Evaluation of Molecular Networks for Discovery of Disease Genes. *Cell systems*, **6**, 484-495 e485.

Supplemental Table 1. Comparison between the two versions of HumanNet

	HumanNet v1	HumanNet v2
**CC**	Based on ~750k Medline abstracts	Based on ~300k full-text articles from PubMed Central
**CE**	Not available	Based on >100 shRNA and >400 CRISPR-Cas9-based essential gene profiles
**CX**	Based on 21 microarray-based GSEs<br>(1,603 samples in total)	Based on 125 microarray-based and 33 RNA-seq-based GSEs<br>(16,220 samples in total)
**DB**	Not available	Based on three pathway databases (KEGG, BioCarta, and Reactome)
**DP**	Domain profiles by InterPro v14.1<br>Profile association was measured by mutual information (MI)	Domain profiles by InterPro v46<br>Profile association was measured by weighted mutual information (WMI)
**GN**	Based on 432 prokaryotic (393 bacteria and 31 archaea) genomes<br>Used probability-based gene neighborhood only	Based on 1748 prokaryotic (1626 bacteria and 122 archaea) genomes, 754 human metagenomes, and 242 ocean sample metagenomes<br>Used probability-, distance-, and metagenome-based gene neighborhoods
**HT**	Based on 1 protein complex mapping data set and 2 binary PPI screen data sets	Based on 7 protein complex mapping data sets and 5 binary PPI screen data sets
**IL**	Transferred 13 outdated networks (7 PPI networks and 6 functional networks) for 3 species (YeastNet v2, WormNet v2, Fly PPIs)<br>All 13 orthology-transferred networks were provided as individual networks.	Transfer 10 latest networks (9 PPI networks and 1 genetic interaction network) for 5 species (YeastNet v3, WormNet v3, FlyNet v1, DanioNet v1, MouseNet v2)<br>Transfer PPIs of four vertebrate species (dog, cattle, rat, and chicken) in iRefIndex.<br>All orthology-transferred networks were integrated into a single network.
**LC**	HPRD, BIND, BIOGRID, IntAct, MINT, and Reactome (outdated databases)	IRefIndex version 14 (includes the latest versions of BIND, BioGRID, CORUM, DIP, HPRD, InnateDB, IntAct, MatrixDB, MINT, MPact, MPIDB, and MPPI databases)
**PG**	Based on concatenated profiles composed of 432 prokaryotic (393 bacteria and 31 archaea) genomes	Based on 1626 bacterial and 122 archaeal genomes<br>Analyzed two phylogenetic profiles for bacteria and Archaea, separately.

CC, co-citation; CE, co-essentiality; CX, co-expression; DB, pathway database; DP, domain profile; GN, gene neighborhood; HT, high throughput PPI; IL, interologs; LC, literature curated PPI; PG, phylogenetic profile

Supplemental Table 2. High-throughput PPI data sets used for HumanNet v2

Publication	PMID	Data type	# Inferred links	# Genes
Nature. 2017 May 25;545(7655):505-509	28514442	Complex	33,501	9,924
Cell. 2015 Oct 22;163(3):712-23	26496610	Complex	21,132	5,061
Nature. 2015 Sep 17;525(7569):339-44	26344197	Complex	14,516	3,246
Cell Rep. 2014 Jul 10;8(1):297-310	24981860	Complex	16,250	2,010
Science. 2010 Apr 30;328(5978):593-9	20360068	Complex	3,994	678
Cell. 2009 Jul 23;138(2):389-403	19615732	Complex	4,185	1,736
Mol Syst Biol. 2007;3:89	17353931	Complex	1,730	1,462
Cell. 2014 Nov 20;159(5):1212-1226	25416956	Binary	6,515	4,135
Mol Syst Biol. 2011 Oct 11;7:536	21988832	Binary	1,668	1,228
Nat Methods. 2011 Jun;8(6):478-80	21516116	Binary	1,704	1,483
Nat Methods. 2009 Jan;6(1):83-90	19060904	Binary	230	223
Nature. 2005 Oct 20;437(7062):1173-8	16189514	Binary	2,444	1,454

Complex, protein complex mapping data; Binary, binary protein-protein interaction screen data

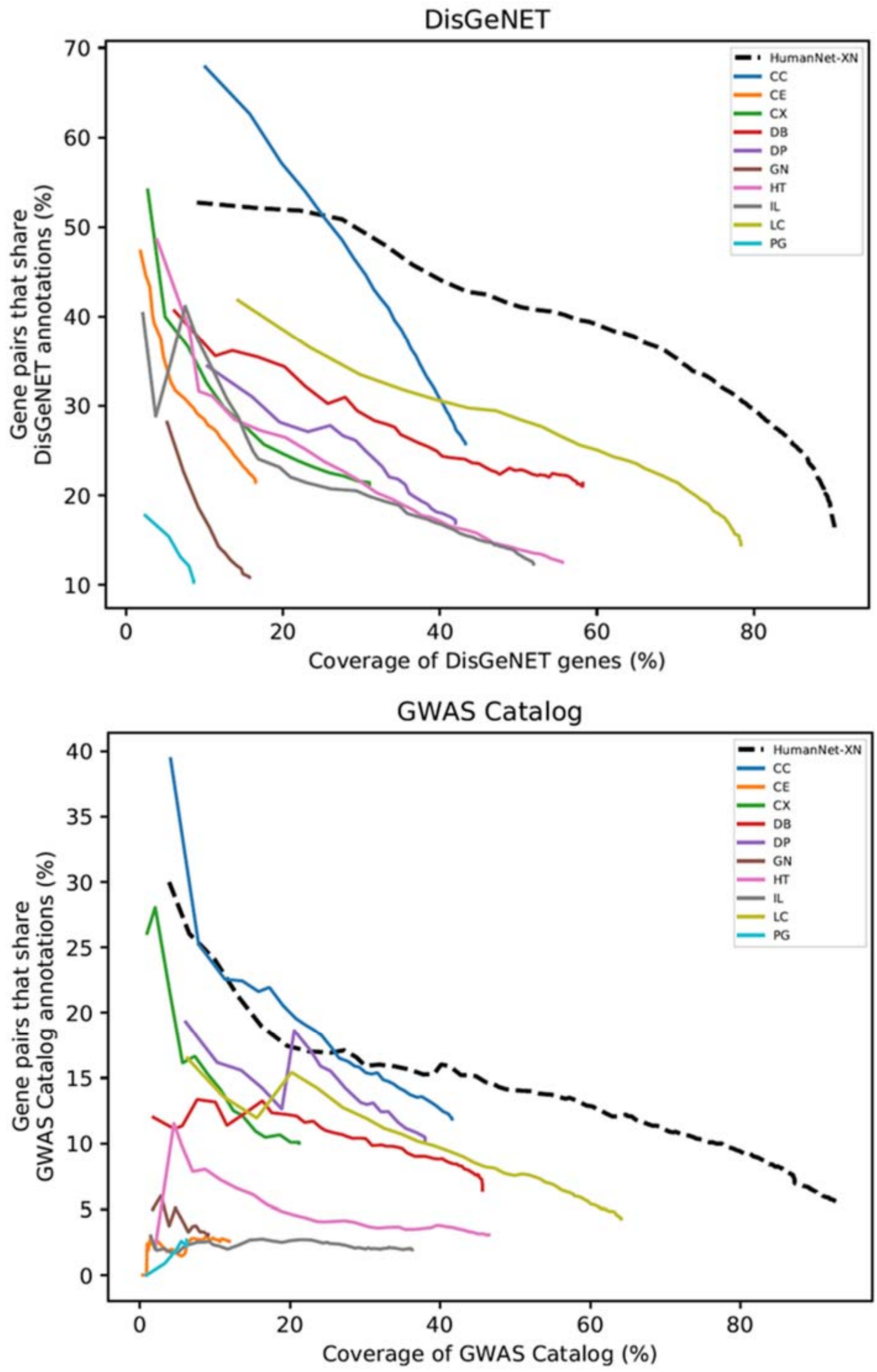

Supplemental Figure 1. Assessment of HumanNet component networks for each evidences based on measuring the precision of identifying gene pairs linked to the same human diseases (defined by DisGeNET or GWAS catalog with timestamp filtration) as a function of the coverage of the database genes.

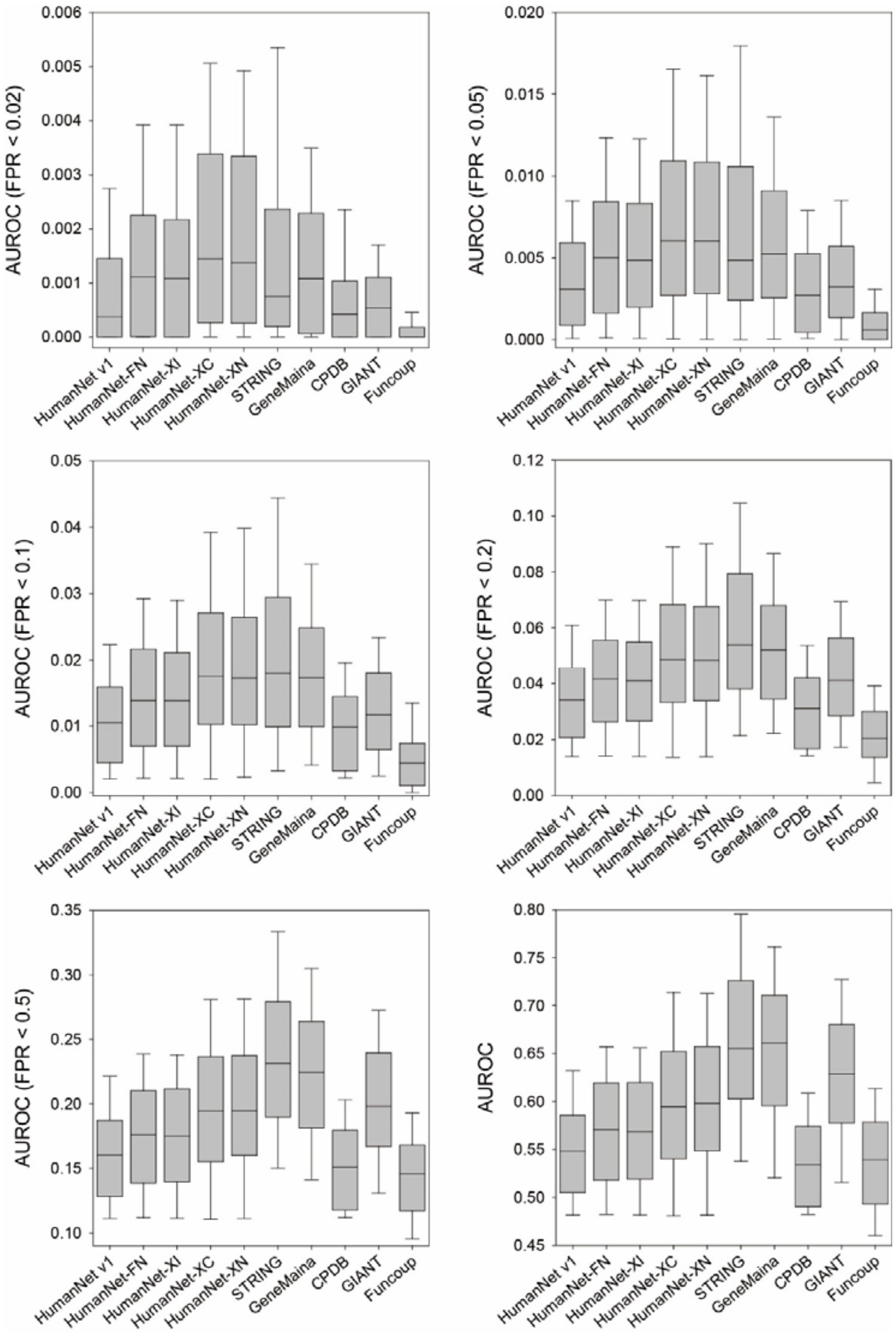

Supplemental Figure 2. Assessment of predictive ability of networks with direct neighborhood approach for unbiased GWAS catalog disease gene sets based on the distribution of the area under receiver operating characteristic curve (AUROC) until different false positive rate (FPR < 0.02, 0.05, 0.1, 0.2, 0.5) and for the entire range. Notably, HumanNet-XC performed best until 5% FP which corresponds to ~1000 most significant candidates for each disease, while STRING, GeneMania, and GIANT performed better for lower ranked predictions.
